# Genetic Variants in Hormone-Related Genes and Risk of Breast Cancer

**DOI:** 10.1371/journal.pone.0069367

**Published:** 2013-07-23

**Authors:** Tess Clendenen, Anne Zeleniuch-Jacquotte, Isaac Wirgin, Karen L. Koenig, Yelena Afanasyeva, Eva Lundin, Alan A. Arslan, Tomas Axelsson, Asta Försti, Göran Hallmans, Kari Hemminki, Per Lenner, Nirmal Roy, Roy E. Shore, Yu Chen

**Affiliations:** 1 Department of Population Health, New York University School of Medicine, New York, New York, United States of America; 2 New York University Cancer Institute, New York University School of Medicine, New York, New York, United States of America; 3 Department of Environmental Medicine, New York University School of Medicine, New York, New York, United States of America; 4 Department of Medical Biosciences, Pathology, Umeå University, Umeå, Sweden; 5 Department of Obstetrics and Gynecology, New York University School of Medicine, New York, New York, United States of America; 6 Molecular Medicine, Department of Medical Sciences, Uppsala University, Uppsala, Sweden; 7 Division of Molecular Genetic Epidemiology, German Cancer Research Center (DKFZ), Heidelberg, Germany; 8 Center for Primary Health Care Research, Clinical Research Center, Lund University, Malmö, Sweden; 9 Department of Public Health and Clinical Medicine/Nutritional Research, Umeå University, Umeå, Sweden; 10 Department of Oncology, Umeå University Hospital, Umeå, Sweden; 11 Radiation Effects Research Foundation, Hiroshima, Japan; University of Modena & Reggio Emilia, Italy

## Abstract

Sex hormones play a key role in the development of breast cancer. Certain polymorphic variants (SNPs and repeat polymorphisms) in hormone-related genes are associated with sex hormone levels. However, the relationship observed between these genetic variants and breast cancer risk has been inconsistent. We conducted a case-control study nested within two prospective cohorts to assess the relationship between specific genetic variants in hormone-related genes and breast cancer risk. In total, 1164 cases and 2111 individually-matched controls were included in the study. We did not observe an association between potential functional genetic polymorphisms in the estrogen pathway, *SHBG* rs6259, *ESR1* rs2234693, *CYP19* rs10046 and rs4775936, and *UGT1A1* rs8175347, or the progesterone pathway, *PGR* rs1042838, with the risk of breast cancer. Our results suggest that these genetic variants do not have a strong effect on breast cancer risk.

## Introduction

Epidemiological evidence indicates a key role for sex hormones in breast cancer development. High circulating levels of estrogens and androgens have been consistently associated with increased breast cancer risk in postmenopausal women [1], [2], [3], [4]. Established breast cancer risk factors, *e.g.,* early age at menarche, nulliparity, late age at menopause, use of estrogen plus progestin hormone replacement therapy, and BMI among postmenopausal women, are thought to affect risk through modulation of sex hormones. Genetic variants in hormone-related genes have been shown to be associated with sex hormone levels [5], [6], [7], [8], [9]. However, associations observed between genetic variants in sex-hormone related genes and breast cancer risk have generally been inconsistent [10], [11], except for two single nucleotide polymorphisms (SNP) near the *ESR1* gene (rs2046210 and rs12662670) that were significantly associated with risk in several genome wide association studies [12], [13], [14], [15], [16], [17]. We assessed whether selected polymorphisms in genes that have been shown to be associated with sex hormone levels or hormone signaling are related to risk of breast cancer.

We selected several genes that encode for proteins involved in hormone signaling and metabolism: sex hormone-binding globulin (*SHBG*), which binds to, and reduces the biological availability of estrogens and androgens; the progesterone receptor (*PGR*); the estrogen receptor alpha (*ESR1*); aromatase (*CYP19*), which converts androgens to estrogens; and UDP-glucuronosyltransferase 1A1 (*UGT1A1*), which glucuronidates estrogens, thereby facilitating their excretion in urine. Candidate polymorphisms in these genes were selected based on their potential functional role (*e.g.,* regulating endogenous hormone levels) and reported association with breast cancer risk at the time of initiation of our study. We selected polymorphisms that were associated with breast cancer risk in some, but not all, studies. The following polymorphisms were genotyped: *SHBG* rs6259 [5], [8], [18], [19], [20]; *PGR* rs1042838 [11], [20], [21], [22], [23], [24], [25]; *ESR1* rs2234693 [20], [26], [27], [28], *CYP19* rs10046 and rs4775936 [5], [7], [20], [29], [30], [31], [32], [33]; and *UGT1A1* rs8175347 [34], [35], [36], [37], [38], [39].

## Methods

### Ethics Statement

The Institutional Review Board of New York University School of Medicine and the Regional Ethical Committee of the University of Umeå, Sweden, reviewed and approved this study. Written informed consent was obtained from all participants at enrollment.

### Study Subjects

We conducted a case-control study nested within two prospective cohorts: the Northern Sweden Mammary Screening Cohort (NSMSC) [40] and the New York University Women’s Health Study (NYUWHS). Details about the parent cohorts and breast cancer case ascertainment has been reported previously [3]. Briefly, the NYWHS cohort includes 14,274 healthy women (ages 34–65) enrolled between 1985–1991 at a mammography screening clinic in New York City and the NSMSC cohort includes over 28,000 healthy women (ages 40–69) enrolled between 1995–2006 during a population-based breast cancer screening program in Västerbotten County, Sweden. For the present study, only women self-described as Caucasian, African American, or Hispanic were included. All incident cases of invasive breast cancer, a total of 1164 cases, were included in our study (658 cases from NYUWHS and 506 cases from NSMSC). Two controls were individually matched to each case. Controls were selected at random from members of the same cohort who were alive and free of cancer at the time of diagnosis of the case, and who matched the case on age at enrollment (±6 months) and date of blood donation (±3 months). NYUWHS cases and controls were also matched on menopausal status. Most of the cases from the NSMSC had at least one control matched on menopausal status (92%). In total, 2111 controls were included in the study (1099 from NYUWHS and 1012 from NSMSC).

### Laboratory Methods

For the NYUWHS participants, DNA was extracted from blood clots or cell precipitates (prepared by centrifugation of whole blood collected at blood donation) for 42% of participants. For the remainder of NYUWHS participants, DNA was extracted from serum. Samples were genotyped using the TaqMan® approach [41], [42] with an ABI 7900 Real-Time PCR instrument (Applied Biosystems, Foster City, CA). The percent of successful genotyping calls was ≥98% for all genetic variants. Prior to the case-control study, a pilot study was conducted to examine genotype concordance across sample types (serum, clots, cell precipitates) for the NYUWHS study. For samples from the same participant (n = 50 subjects with all three sample types plus n = 68 subjects with two sample types), genotype concordance was ≥99%.

For the NSMSC participants, DNA was isolated from buffy coats. Genotyping was performed at the SNP Technology Platform at Uppsala University Hospital (www.genotyping.se) for five SNPs (rs4775936, rs10046, rs6259, rs2234693, rs1042838). Four of these SNPs (rs4775936, rs10046, rs6259, rs2234693) were assayed using the GenomeLab SNPStream 12plex-system (Beckman Coulter) and one (rs1042838) using the FP-TDI system. *UGT1A1* rs8175347 was assayed at the German Cancer Research Center in Heidelberg using fluorescent fragment analysis on an ABI PRISM 3100 Genetic analyzer with the GeneMapper software version 3.0 (Applied Biosystems). The percentage of samples with successful calls was ≥98% for all polymorphisms. We conducted a pilot study to assess genotype concordance across duplicate samples from NSMSC participants (n = 164 duplicates). The concordance between samples from the same participant was ≥99% for all loci.

For both cohorts, each case and her individually-matched controls were analyzed as a set on the same 96-well plate. Quality control samples (10%) were included on each plate and were interspersed throughout the plate with the case-control samples. Laboratory personnel were blinded as to case-control status and the identity of the quality control samples.

### Statistical Methods

We assessed deviation from Hardy-Weinberg equilibrium (HWE) in each cohort for each genetic variant with a chi-square test. Odds ratios and 95% confidence intervals for breast cancer risk were estimated using the conditional logistic regression model, as appropriate for the matched study design. All models were adjusted for race/ethnicity (Caucasian, African American, or Hispanic) and through matching, were also adjusted for age at blood donation, duration of sample storage, and menopausal status. Multivariate-adjusted models included other known risk factors for breast cancer: family history of breast cancer (in a first degree relative), age at menarche, age at first birth/parity (≤20 years, 21–25 years, 26–30 years, >30 years, nulliparous), ever use of hormone replacement therapy, and body mass index (BMI). For covariates with missing data (age at menarche, age at first birth, use of hormone replacement therapy, and BMI), we performed multiple imputation of missing data for each cohort separately using a fully conditional specification model [43] including family history of breast cancer and case-control status along with the imputed variables. Each of the imputed variables had <4% missing data. We also conducted a logistic regression including all of the estrogen-related variants simultaneously (all variants except PGR-12), where homozygous genotypes for the variant associated with higher estrogen was coded as one and the other genotypes were coded as zero and the sum of the scores was modeled as the independent variable (women with four or five variants were grouped because there were only 3 cases and 4 controls who had five high estrogen variants). Heterogeneity between cohorts was assessed by comparing models with cross-product terms (cohort×genotype) to models excluding them using the likelihood ratio test. Analyses were also conducted separately for Caucasians (n = 1067 cases, 1931 controls) and for estrogen receptor positive (ER+) breast cancer (n = 625 cases, 1091 controls).

## Results

Descriptive statistics for the cases and controls are shown in Table 1. The expected relationships between breast cancer and the traditional risk factors were observed. Among postmenopausal women, cases had a higher mean BMI than controls. Cases were more likely than controls to be nulliparous, to have ever used HRT, and to have a family history of breast cancer. Among parous women, average age at first birth was greater for cases than controls. The frequency of genotypes within controls did not deviate from Hardy-Weinberg Equilibrium by cohort (all p-values >0.05).

**Table 1:** Distributions of demographic and lifestyle variables by breast cancer status.

Demographic and Lifestyle Variables	Cases	Controls	p-value^c^
	(N = 1164)	(N = 2111)	
Age (years) at enrollment, mean (SD)	54.5 (8.2)	54.9 (8.1)	Matched
Age (years) at menarche, mean (SD)	12.8 (1.5)	12.9 (1.5)	0.12
Unknown	17	44	
Age (years) at diagnosis, mean (SD)	61.9 (8.5)	–	
BMI (kg/cm^2^) at enrollment, mean (SD)			
Pre-menopausal	24.3 (4.1)	24.7 (4.5)	0.14
Post-menopausal	26.0 (4.4)	25.3 (4.1)	<0.01
Unknown	29	48	
Menopausal status at enrollment, n, %			Matched
Pre-menopausal	442 (38.0)	745 (35.3)	
Post-menopausal	722 (62.0)	1366 (64.7)	
Nulliparous at enrollment, n, %	261 (23.2)	367 (17.8)	<0.01
Unknown	38	54	
Age (years) at first full-term pregnancy, mean (SD)^a^	25.4 (4.8)	24.8 (4.6)	0.01
Unknown	22	34	
Race/ethnic background, n, %			0.47
Caucasian	1067 (91.7)	1931 (91.5)	
African American	72 (6.2)	117 (5.5)	
Hispanic	25 (2.2)	63 (3.0)	
Ever users of HRT, n, %^b^	393 (35.6)	648 (31.7)	0.03
Unknown	60	66	
Family history of breast cancer, n, %	218 (18.7)	306 (14.5)	<0.01
Tumor Receptor Status			
ER- positive	625 (72.7)	–	
ER- negative	235 (27.3)	–	
Unknown	304		

a For parous women only.

b HRT use during follow-up (for NYUWHS) or use history assessed at enrollment (NSMSC).

c p-values were estimated using logistic regression conditional on matching sets.

Table 2 shows that there were no statistically significant associations between the selected genetic variants in hormone-related genes and risk of breast cancer in age- or multivariate-adjusted models. For each genotype, variants are listed in order of expected increasing estrogen (or progesterone for the PGR SNP) exposure. There was no association with risk for individuals with multiple genotypes associated with high estrogen levels. Tests for heterogeneity by cohort were not significant. The odds ratios were not appreciably different in analyses restricted to Caucasians (data not shown). Tests for interaction between each genetic polymorphism and age at diagnosis were not significant. ORs were not significant and were generally similar in magnitude and direction for analyses restricted to ER+ breast cancer (data not shown), except that the OR estimates were no longer greater than one for the CYP19 rs10046 TT and rs4775936 AA genotypes. For SHBG rs6259, the OR was somewhat greater for ER+ tumors (OR for GG vs. GA/AA: 1.22, 95% CI: 0.94–1.60) than for all tumors combined (for GG vs. GA/AA: 1.02, 95% CI: 0.84–1.23), though the association was not significant.

**Table 2:** Associations between genetic variants in hormone-related genes and breast cancer risk in pre- and post-menopausal women.

	*n* (Cases/Controls)	Ethnicity-adjusted	Multivariate-adjusted
		ORs (95% CI)^a^	ORs (95% CI)^b^
*SHBG* (rs6259)			
AA	15/28	1.00	1.00
GA	197/351	1.00 (0.53–1.92)	0.98 (0.51–1.89)
GG	933/1655	1.02 (0.54–1.91)	0.99 (0.52–1.89)
p-trend		0.90	0.89
GG vs. GA/AA		1.01 (0.84–1.22)	1.02 (0.84–1.23)
*PGR-12* (rs1042838)			
TT	26/54	1.00	1.00
GT	288/523	1.14 (0.70–1.86)	1.21 (0.74–1.98)
GG	846/1516	1.20 (0.74–1.93)	1.25 (0.77–2.02)
p-trend		0.42	0.44
GG vs. GT/TT		1.06 (0.90–1.25)	1.05 (0.89–1.24)
*ESR1* (rs2234693)			
TT	334/660	1.00	1.00
CT	585/1010	1.14 (0.96–1.35)	1.13 (0.95–1.34)
CC	244/436	1.10 (0.90–1.35)	1.11 (0.90–1.36)
p-trend		0.28	0.28
*CYP19 3′UTR* (rs10046)			
CC	306/549	1.00	1.00
CT	548/1032	0.95 (0.80–1.13)	0.94 (0.79–1.12)
TT	308/523	1.07 (0.87–1.31)	1.04 (0.85–1.28)
p-trend		0.53	0.71
*CYP19 5′Flank* (rs4775936)			
GG	361/654	1.00	1.00
GA	531/1011	0.94 (0.79–1.12)	0.94 (0.79–1.12)
AA	271/438	1.13 (0.92–1.39)	1.10 (0.89–1.36)
p-trend		0.33	0.46
*UGT1A1* (rs8175347)			
6/6	510/938	1.00	1.00
6/7	478/846	1.03 (0.88–1.21)	1.06 (0.90–1.24)
7/7	151/257	1.06 (0.84–1.34)	1.06 (0.84–1.34)
p-trend		0.60	0.51
Combined model for estrogen pathway variants^c^			
0 high E genotypes	108/190	1.00	1.00
1 high E genotypes	485/911	0.93 (0.72, 1.21)	0.93 (0.71, 1.21)
2 high E genotypes	267/441	1.07 (0.81, 1.41)	1.08 (0.82, 1.44)
3 high E genotypes	194/310	1.12 (0.83, 1.50)	1.11 (0.82, 1.50)
4–5 high E genotypes	64/106	1.06 (0.71, 1.57)	1.00 (0.67, 1.50)
p-trend		0.15	0.21

a Models were adjusted for ethnicity (Caucasian, African American, Hispanic) and through matching, were also adjusted for age at blood donation, duration of sample storage, and menopausal status.

b Models were adjusted for ethnicity (Caucasian, African American, Hispanic), age at first birth/parity (≤20 years, 21–25 years, 26–30 years, >30 years, nulliparous), age at menarche, family history of breast cancer, ever use of HRT, and BMI, and through matching, were also adjusted for age at blood donation, duration of sample storage, and menopausal status.

c For each genetic variant (except *PGR-12* rs1042838), the genotype associated with higher estrogen exposure (see below) was assigned a value of 1 and other genotypes (homozygous and heterozygous for the lower estrogen exposure allele) were assigned 0. A score was created by adding the values. Women with four or five high estrogen variables were grouped because there were too few women with five high estrogen variables to assess separately (3 cases/4 controls).

**Notes: For each genotype, variants are listed in order of expected increasing estrogen (or progesterone for** ***PGR-12*** **) exposure:**

*SHBG* (rs6259) A allele is associated with higher SHBG levels. SHBG binds to estrogens and reduces their bioavailability. G allele = higher estrogen exposure.

*PGR-12* (rs1042838) T allele has been shown to reduce the *PGR* transcript stability and the response to progesterone. G allele = higher progesterone exposure.

*ESR1* (rs2234693) C allele may be associated with increased ERα transcription. C allele = possible higher exposure to estrogen signaling.

*CYP19* 3′UTR (rs10046) T allele is associated with increased transcriptional activity of aromatase (converts androgens to estrogens). T allele = higher estrogen exposure.

*CYP19* 5′Flank (rs4775936) A allele is associated with increased transcriptional activity of aromatase (converts androgens to estrogens). A allele = higher estrogen exposure.

*UGT1A1* (rs8175347) 7 repeat allele is associated with reduced glucuronidation (and clearance) of estrogens. 7 repeat allele = higher estrogen exposure.

## Discussion

Our results are in agreement with those of large meta-analyses of hormone-related genetic variants and breast cancer risk (which included over 10,000 cases and 10,000 controls) that did not observe an association for *SHBG* rs6259, *PGR* rs1042838, *CYP19* rs10046 and rs4775936, or *UGT1A1* rs8175347 variants [32], [44]. Two meta-analyses (both with over 10,000 cases and 10,000 controls), observed a borderline inverse association for *ESR1* rs2234693 (OR for C vs. T: 0.97, 95% CI: 0.93–1.00 [32], p = 0.055 and OR for CC vs. TT: 0.92, 95% CI: 0.86–0.99 [28]), which we did not observe in our study.

Three of the genetic variants we selected directly influence estrogen levels: *CYP19* rs10046 and rs4775936 and *UGT1A1* rs8175347. *CYP19* encodes for the enzyme aromatase, which converts androgens to estrogens. The rs10046 T allele and the rs4775936 A allele have been shown to be associated with higher levels of circulating estrogen or estrogen to androgen ratios in several studies [5], [7], [29], [45], including our own [46]. The 7 base repeat allele of *UGT1A1* rs8175347 is associated with lower transcriptional activity and may result in reduced glucuronidation of estrogens [34]. Circulating estrogen levels were higher among women with 7 base repeat alleles in our study [46] as in most other studies [34], [47]. However, despite the relationship between these SNPs and estrogen levels, most studies, including our own, have not observed any association between these SNPs in *CYP19* and *UGT1A1* and breast cancer risk [32], [48].

*SHBG* rs6259 influences estrogen bioavailability and the A allele is associated with higher SHBG levels (suggesting lower estrogen bioavailability) in most [5], [8], [18], [19], [49], but not all studies [6], [50]. Consistent with most previous studies [32], we did not observe an association between rs6259 and risk of breast cancer.

*PGR* rs1042838 influences progesterone signaling and the T allele is in high linkage disequilibrium with the PROGINS allele, which reduces the *PGR* transcript stability and may decrease the response to progesterone [23]. We did not observe an association between *PGR* rs1042838 and breast cancer risk, in agreement with nearly all previous studies [32].

The function of the *ESR1* rs2234693 SNP has not been clearly demonstrated, though the C allele produces a binding site for the B-myb transcription factor, which may result in an alternative form and/or altered expression of the estrogen receptor, and thus influence estrogen signaling [26]. Two large meta-analyses reported a moderate reduction in breast cancer risk associated with the C allele [28], [32]. We did not observe any significant association for *ESR1* rs2234693, consistent with the findings of most other studies (reviewed in [28], [32]). We note that *ESR1* rs2234693 is not in linkage disequilibrium with the SNPs near the ESR1 gene that were associated with breast cancer risk in several genome wide association studies [12], [13], [14], [15], [16], [17].

There is substantial evidence that higher levels of circulating estrogens are associated with an increased risk of breast cancer in postmenopausal women. A pooled analysis of nine prospective epidemiologic studies found that for a doubling in estradiol levels, there was about a 29% increase in risk [51]. The genetic polymorphisms we examined were associated with an increase in estrone levels ranging from 6–28% in our study, which is consistent with observations from other studies for estradiol (range ∼5–60%) [5], [7], [29], [34], [45], [47]. Thus, it is unlikely that the effect of any of these SNPs alone on estrogen levels is large enough to have a measurable effect on breast cancer risk and may explain why most studies, including our own, did not find an association with risk.

The ages at enrollment into the parent cohorts were 34–65 for the NYUWHS and 40–69 for the NSMSC. While generalizability of our results to other populations requires caution, it is unlikely that the relationship between these common genetic variants and breast cancer risk is limited to women in the same age range as in our study.

In agreement with the largest meta-analyses of epidemiological studies to date, we did not observe an association for *SHBG* rs6259, *PGR* rs1042838, *ESR1* rs2234693, *CYP19* rs10046 and rs4775936, and *UGT1A1* rs8175347 and breast cancer risk. The effects of other genetic variants in these or other hormone-related genes, individually or in combination, may have an effect on breast cancer risk. In conclusion, our study provides supportive evidence that these genetic variants in hormone-related genes are not likely to have a strong effect on breast cancer risk.
